# Current epidemiological profile and characteristics of traumatic and nontraumatic spinal cord injury in Jinan, China: A two-center retrospective observational study

**DOI:** 10.1097/MD.0000000000051397

**Published:** 2026-11-06

**Authors:** Linyang Song, Kun Wang, Min Zhao, Xiaolin Chen, Hezi Chang, Yingrun Fu, Song Ruoxian

**Affiliations:** a Department of Orthopaedics, Qilu Hospital of Shandong University, Jinan, China; b Department of Orthopaedics, Affiliated 960th Hospital of PLA, Shandong Second Medical University, Jinan, China.

**Keywords:** epidemiology, Jinan, nontraumatic spinal cord injury, prevention, traumatic spinal cord injury

## Abstract

Spinal cord injury (SCI) is a highly disabling neurological condition associated with long-term functional impairment and substantial healthcare needs. In Jinan, hospital-based evidence directly comparing traumatic SCI (TSCI) and nontraumatic SCI (NTSCI) within the same regional tertiary-care system remains limited. This study aimed to describe and compare their epidemiological and clinical profiles to inform targeted prevention and hospital-based care planning. We conducted a 2-center, hospital-based retrospective observational study of patients with SCI admitted between 2015 and 2024. Categorical variables were summarized as n (%) and compared between TSCI and NTSCI using Pearson chi-square tests or Fisher–Freeman–Halton exact tests, as appropriate. Because length of stay was markedly right-skewed, it was summarized as median (interquartile range [IQR]) and compared using the Mann–Whitney *U* test. All comparisons were two-sided and unadjusted. A total of 1334 patients with SCI were included (1134 TSCI and 200 NTSCI). TSCI showed a greater male predominance than NTSCI (78.66% vs 56.50%, *P* < .001), and age-group distributions also differed (*P* < .001). Falls (46.03%) and traffic accidents (40.65%) were the leading TSCI mechanisms; electric bicycle-related crashes accounted for the largest share of traffic-related TSCI (191/461, 41.43%). The leading NTSCI etiologies were neoplasm (33.00%), demyelinating disease (20.00%), infectious disease (17.00%), vascular disease (15.50%), and degenerative disease (14.50%). American Spinal Injury Association Impairment Scale distributions differed between groups (*P* < .001). Median length of stay was 12 days (IQR = 8–21) for TSCI and 46 days (IQR = 33.75–61) for NTSCI (*P* < .001). A primary in-hospital complication was recorded in 78.31% of TSCI and 80.00% of NTSCI patients (*P* = .591); however, the distribution of primary complication categories differed overall (*P* = .040). Urinary tract infection was more frequent in TSCI (24.78% vs 16.00%, *P* = .007), whereas pulmonary infection was more frequent in NTSCI (12.26% vs 18.00%, *P* = .027). Within these 2 tertiary hospitals in Jinan, TSCI and NTSCI showed different demographic, neurological, treatment, hospitalization, and primary complication patterns. TSCI was characterized by prominent fall- and traffic-related injury mechanisms, whereas NTSCI showed heterogeneous etiologies and substantially longer hospitalization. These hospital-based findings support targeted prevention priorities and differentiated clinical care pathways but should not be interpreted as population-level incidence estimates or as independent effects of traumatic versus nontraumatic etiology.

## 1. Introduction

Spinal cord injury (SCI) is a highly disabling neurological condition that imposes substantial societal and economic burdens, leading to persistent functional impairment and increased healthcare needs.^[1,2]^ At present, China lacks a national SCI registry, and regional evidence describing long-term trends and directly comparing the epidemiological and clinical profiles of traumatic SCI (TSCI) and nontraumatic SCI (NTSCI) remains limited. Given the pronounced heterogeneity between TSCI and NTSCI in etiologic composition, onset mechanisms, diagnostic pathways, and clinical management, pooling these entities in a single analysis may obscure clinically meaningful distinctions. Therefore, regional hospital-based investigations that explicitly categorize and compare TSCI and NTSCI are important for characterizing case mix and informing prevention and care-planning priorities.

Previous studies have demonstrated substantial geographic variation in the etiologic spectrum, onset patterns, and clinical pathways for both TSCI and NTSCI.^[2]^ In general, TSCI is predominantly attributable to external traumatic events such as traffic accidents and falls,^[1,3–7]^ whereas NTSCI more commonly arises from degenerative disorders, neoplasms, infections/inflammation, vascular etiologies, and immune-mediated diseases.^[5,7,8]^ These differences underscore the need for region-specific stratified descriptions and parallel comparisons between TSCI and NTSCI. While international studies describing either TSCI or NTSCI are relatively abundant, investigations that simultaneously distinguish and compare both types within the same geographic area and time window remain limited. For example, in South Korea, falls account for the largest share of TSCI (56.4%), followed by traffic accidents (32.6%).^[9]^ In Beijing, China, falls from height are the leading cause of TSCI (30.8%), followed by traffic accidents (27.6%) and falls from low height (25.1%).^[10]^ For NTSCI, degenerative disorders and neoplasms are frequently reported in developed settings, whereas neoplasms and infections remain prominent causes in developing settings.^[11]^ However, systematic hospital-based comparative evidence regarding treatment patterns, length of stay (LOS), and in-hospital complications for TSCI versus NTSCI remains insufficient in Jinan.

Accordingly, this study systematically describes and compares demographic characteristics, etiologic spectrum, injury level and neurological severity (American Spinal Injury Association Impairment Scale [AIS]), treatment patterns, LOS, and primary in-hospital complications among hospitalized patients with TSCI and NTSCI from 2 tertiary centers in Jinan between 2015 and 2024. The aim was to provide hospital-based evidence to support targeted prevention priorities and clinical service planning within the regional tertiary-care setting.

## 2. Materials and methods

### 2.1. Study design and setting

This dual-center, hospital-based retrospective observational study included patients with a confirmed diagnosis of SCI who were first admitted to either participating hospital between January 1, 2015, and December 31, 2024. The study sites were the 960th Hospital of the People’s Liberation Army and Qilu Hospital of Shandong University, both class A tertiary hospitals located in Jinan, China. To avoid duplicate counting, for patients transferred between hospitals or admitted multiple times, only the first admission record was retained; inter-hospital referrals for the same patient were merged and treated as a single admission.

### 2.2. Participants and case ascertainment

This study followed the Strengthening the Reporting of Observational Studies in Epidemiology reporting guidelines. Cases were identified using SCI-related International Classification of Diseases, Tenth Revision diagnostic codes and relevant TSCI codes, applying a 2-investigator independent extraction strategy with a 10% randomized cross-check for quality control. Classification of TSCI versus NTSCI and assignment of etiologic categories were cross-verified, with discrepancies adjudicated by a third investigator. Missingness was assessed separately for each analytic variable; no imputation was performed. TSCI was defined as SCI caused by a clearly documented traumatic event (e.g., traffic accident, high fall, or low fall), whereas NTSCI was defined as SCI attributable to nontraumatic etiologies (e.g., neoplasm, demyelinating disease, infection, vascular disease, or degeneration). Standardized variables and data elements were recorded in accordance with the International Spinal Cord Injury Core Data Set (version 1.1). Eligible patients were those hospitalized at either institution during the study period with a confirmed diagnosis of TSCI or NTSCI supported by inpatient medical records. Exclusion criteria were isolated spinal fractures without spinal cord/cauda equina injury; isolated disc herniation without imaging evidence of or resulting in spinal cord/cauda equina syndrome; missing neurological function records that could not be supplemented from original charts; unclear diagnosis or iatrogenic SCI resulting from perioperative complications; and concomitant severe traumatic brain injury or serious medical conditions rendering neurological assessment unreliable, or cases in which SCI management was not the primary treatment objective.

### 2.3. Variables and definitions

Data were collected across multiple domains, including demographic characteristics (age, sex, educational attainment, occupation, marital status, and ethnicity), injury characteristics (admission AIS grade, injury level, and neurological phenotype), etiologic categories, treatments, LOS, and primary in-hospital complications. Complete injury was defined as AIS A and incomplete injury as AIS B to D. Neurological phenotype was derived from injury level and AIS grade according to International Standards for Neurological Classification of Spinal Cord Injury definitions; cervical involvement was classified as tetraplegia and injuries without cervical involvement as paraplegia. TSCI etiologies were classified as traffic accidents, low falls (<1 m), high falls (≥1 m), machinery-related injuries, sports-related injuries, and falling-object injuries. Traffic accidents were further categorized as 4-wheeled motor vehicles, 2-wheeled motor vehicles, electric bicycles, bicycles, and pedestrian events. NTSCI etiologies were classified as demyelinating disease, neoplasm, degenerative disease, vascular disease, and infectious disease. Treatment variables comprised surgery, rehabilitation therapy, traditional therapy, hyperbaric oxygen therapy, assistive device use, and medication. The dataset captured 1 primary in-hospital complication per patient, identified from clinical records, laboratory results, imaging reports, and discharge diagnoses. Primary complication categories were urinary tract infection, pulmonary infection, hyponatremia, pressure ulcer, deep vein thrombosis, and other complications; patients without a recorded primary complication were classified as having no primary in-hospital complication.

### 2.4. Statistical analysis

Continuous age data from the original study database are presented as mean ± standard deviation, and age was additionally categorized into predefined groups for between-group comparison. Categorical variables were summarized as n (%). Between-group comparisons of categorical variables used Pearson chi-square tests; Fisher–Freeman–Halton exact tests with Monte Carlo estimation were used for sparse multi-category contingency tables. LOS was markedly right-skewed and was therefore summarized as median (interquartile range [IQR]); TSCI and NTSCI LOS distributions were compared using two-sided Mann–Whitney *U* tests. A two-sided *P* < .05 was considered statistically significant. All comparisons were unadjusted and were intended to characterize between-group patterns rather than estimate independent causal effects.

### 2.5. Ethics

The study protocol was approved by the Ethics Committees of the 960th Hospital of the People’s Liberation Army and Qilu Hospital of Shandong University (approval numbers: No. 2025085 and No. KYLL-202506-029-1). Informed consent was waived due to the retrospective design and minimal risk. All data were de-identified after extraction and used solely for research purposes.

## 3. Results

### 3.1. Patient selection

A total of 1417 patients with SCI were initially identified between 2015 and 2024. During screening, 83 cases were excluded for the following reasons: severe traumatic brain injury precluding reliable neurological assessment (n = 31), spinal fractures without associated SCI (n = 29), severe heart disease (n = 4), unclear diagnoses (n = 6), and incomplete medical records or questionable diagnoses (n = 13). Ultimately, 1334 patients were included in the final analysis. There were no missing values for the key analytic variables used in the revised comparisons, including etiologic classification, AIS grade, injury level, LOS, surgery, and primary complication classification. Accordingly, analysis-specific denominators were 1134 for TSCI and 200 for NTSCI for these variables.

### 3.2. Baseline characteristics

The final cohort comprised 1334 patients, including 1134 with TSCI and 200 with NTSCI. The TSCI group included 892 males (78.66%) and 242 females (21.34%), whereas the NTSCI group included 113 males (56.50%) and 87 females (43.50%); the sex distribution differed between groups (*P* < .001). The overall mean age was 60.56 ± 17.10 years (TSCI: 62.00 ± 18.60 years; NTSCI: 52.40 ± 19.30 years). Patients aged 61 to 75 years represented the largest age category overall (34.33%) and within both TSCI (33.25%) and NTSCI (40.50%); age-group distributions differed between groups (*P* < .001). Most patients had elementary or middle school education (64.09%, 855/1334), and farmers constituted the largest occupational group (42.20%, 563/1334). Additional demographic characteristics and unadjusted group comparisons are presented in Table 1.

**Table 1:** Baseline characteristics of patients with TSCI and NTSCI, 2015 to 2024.

Variable	TSCI (n = 1134)	NTSCI (n = 200)	Overall (n = 1334)	*P* value
Age, yr (mean ± SD)	62.00 ± 18.60	52.40 ± 19.30	60.56 ± 17.10	
Age group, yr				<.001
0–15	35 (3.09%)	12 (6.00%)	47 (3.52%)	
16–30	54 (4.76%)	20 (10.00%)	74 (5.55%)	
31–45	104 (9.17%)	29 (14.50%)	133 (9.97%)	
46–60	224 (19.75%)	49 (24.50%)	273 (20.46%)	
61–75	377 (33.25%)	81 (40.50%)	458 (34.33%)	
≥76	340 (29.98%)	9 (4.50%)	349 (26.16%)	
Sex				<.001
Male	892 (78.66%)	113 (56.50%)	1005 (75.34%)	
Female	242 (21.34%)	87 (43.50%)	329 (24.66%)	
Education level				<.001
Illiterate	1 (0.09%)	5 (2.50%)	6 (0.45%)	
Preschool	3 (0.26%)	1 (0.50%)	4 (0.30%)	
Elementary school	356 (31.39%)	44 (22.00%)	400 (29.99%)	
Middle school	416 (36.68%)	39 (19.50%)	455 (34.11%)	
High school	278 (24.51%)	65 (32.50%)	343 (25.71%)	
College or more	80 (7.05%)	46 (23.00%)	126 (9.45%)	
Occupation				<.001
Student	27 (2.38%)	17 (8.50%)	44 (3.30%)	
Farmer	487 (42.95%)	76 (38.00%)	563 (42.20%)	
Worker	168 (14.81%)	30 (15.00%)	198 (14.84%)	
Government officer	28 (2.47%)	12 (6.00%)	40 (3.00%)	
Soldier	7 (0.62%)	3 (1.50%)	10 (0.75%)	
Retired	46 (4.06%)	46 (23.00%)	92 (6.90%)	
Others	371 (32.72%)	16 (8.00%)	387 (29.01%)	
Marital status				<.001
Married	1057 (93.21%)	157 (78.50%)	1214 (91.00%)	
Unmarried	60 (5.29%)	38 (19.00%)	98 (7.35%)	
Divorced	1 (0.09%)	2 (1.00%)	3 (0.22%)	
Widowed	16 (1.41%)	3 (1.50%)	19 (1.42%)	
Ethnicity				<.001
Han	1127 (99.38%)	167 (83.50%)	1294 (97.00%)	
Hui	3 (0.26%)	7 (3.50%)	10 (0.75%)	
Manchu	2 (0.18%)	0 (0.00%)	2 (0.15%)	
Mongolian	2 (0.18%)	2 (1.00%)	4 (0.30%)	
Miao	0 (0.00%)	4 (2.00%)	4 (0.30%)	
Tujia	0 (0.00%)	4 (2.00%)	4 (0.30%)	
Zhuang	0 (0.00%)	14 (7.00%)	14 (1.05%)	
Hakka	0 (0.00%)	1 (0.50%)	1 (0.07%)	
Yao	0 (0.00%)	1 (0.50%)	1 (0.07%)	
AIS grade				<.001
A	387 (34.13%)	11 (5.50%)	398 (29.84%)	
B	250 (22.05%)	46 (23.00%)	296 (22.19%)	
C	244 (21.52%)	73 (36.50%)	317 (23.76%)	
D	253 (22.31%)	70 (35.00%)	323 (24.21%)	
Neurological phenotype				<.001
Tetraplegia	967 (85.27%)	73 (36.50%)	1040 (77.96%)	
Paraplegia	167 (14.73%)	127 (63.50%)	294 (22.04%)	
Injury level				<.001
Cervical cord	953 (84.04%)	50 (25.00%)	1003 (75.19%)	
Thoracic cord	89 (7.85%)	56 (28.00%)	145 (10.87%)	
Lumbar cord	42 (3.70%)	49 (24.50%)	91 (6.82%)	
Sacral cord	2 (0.18%)	0 (0.00%)	2 (0.15%)	
Cervicothoracic cord	13 (1.15%)	23 (11.50%)	36 (2.70%)	
Thoracolumbar cord	34 (3.00%)	22 (11.00%)	56 (4.20%)	
Cervico-thoraco-lumbar cord	1 (0.09%)	0 (0.00%)	1 (0.07%)	

Values are n (%) unless otherwise indicated; continuous age data are presented as mean ± SD. Percentages use the corresponding group total as the denominator.

*P* values compare TSCI and NTSCI. Pearson chi-square tests were used for categorical variables with adequate expected counts; Fisher–Freeman–Halton exact tests with Monte Carlo estimation were used for sparse multi-category tables. Age was compared using the predefined age groups.

Neurological phenotype was derived from anatomical injury level (tetraplegia for cervical involvement; paraplegia otherwise).

AIS = American Spinal Injury Association Impairment Scale, NTSCI = nontraumatic spinal cord injury, SD = standard deviation, TSCI = traumatic spinal cord injury.

### 3.3. Etiology

Among the 1134 patients with TSCI, falls were the most common mechanism (low falls: 25.84%, 293/1134; high falls: 20.19%, 229/1134; combined: 46.03%), followed by traffic accidents (40.65%, 461/1134; Table 2). Within traffic-related TSCI, electric bicycle-related crashes were the largest category (191/461, 41.43%), followed by 4-wheeled motor vehicles (173/461, 37.53%), 2-wheeled motor vehicles (48/461, 10.41%), pedestrian events (33/461, 7.16%), and bicycles (16/461, 3.47%; Fig. 1). In the 61- to 75-year TSCI subgroup, falls accounted for 172/377 cases (45.62%) and traffic accidents for 161/377 (42.71%; Table S1, Supplemental Digital Content 1).

**Table 2:** Etiology and neurological phenotype of TSCI and NTSCI.

Etiology	Total, n (%)	Age, yr (mean ± SD)	Complete tetraplegia	Incomplete tetraplegia	Complete paraplegia	Incomplete paraplegia
Traffic accidents	461 (40.65%)	62.80 ± 17.30	153 (33.19%)	270 (58.57%)	20 (4.34%)	18 (3.90%)
Low fall	293 (25.84%)	67.40 ± 16.10	35 (11.95%)	235 (80.20%)	8 (2.73%)	15 (5.12%)
High fall	229 (20.19%)	57.20 ± 20.00	85 (37.12%)	74 (32.31%)	38 (16.59%)	32 (13.97%)
Machinery-related injury	26 (2.29%)	58.90 ± 20.90	9 (34.62%)	13 (50.00%)	3 (11.54%)	1 (3.85%)
Sport	67 (5.91%)	46.00 ± 23.10	9 (13.43%)	42 (62.69%)	4 (5.97%)	12 (17.91%)
Falling objects	58 (5.11%)	62.30 ± 18.00	14 (24.14%)	28 (48.28%)	9 (15.52%)	7 (12.07%)
TSCI overall	1134 (100.00%)	62.00 ± 18.60	305 (26.90%)	662 (58.38%)	82 (7.23%)	85 (7.50%)
Demyelinating disease	40 (20.00%)	40.90 ± 16.90	1 (2.50%)	19 (47.50%)	0 (0.00%)	20 (50.00%)
Neoplasm	66 (33.00%)	51.60 ± 19.80	0 (0.00%)	25 (37.88%)	2 (3.03%)	39 (59.09%)
Degenerative disease	29 (14.50%)	59.10 ± 15.90	1 (3.45%)	17 (58.62%)	1 (3.45%)	10 (34.48%)
Vascular disease	31 (15.50%)	56.30 ± 15.00	0 (0.00%)	6 (19.35%)	1 (3.23%)	24 (77.42%)
Infectious disease	34 (17.00%)	58.70 ± 20.10	0 (0.00%)	4 (11.76%)	5 (14.71%)	25 (73.53%)
NTSCI overall	200 (100.00%)	52.40 ± 19.30	2 (1.00%)	71 (35.50%)	9 (4.50%)	118 (59.00%)

Values are n (%) unless otherwise indicated; continuous age data are presented as mean ± SD. Percentages in neurological phenotype columns are calculated within each etiology.

Complete injury was defined as AIS A and incomplete injury as AIS B to D. Tetraplegia/paraplegia was derived from the anatomical injury level according to the ISNCSCI-based definition.

AIS = American Spinal Injury Association Impairment Scale, ISNCSCI = International Standards for Neurological Classification of Spinal Cord Injury, NTSCI = nontraumatic spinal cord injury, SD = standard deviation, TSCI = traumatic spinal cord injury.

**Figure 1.**
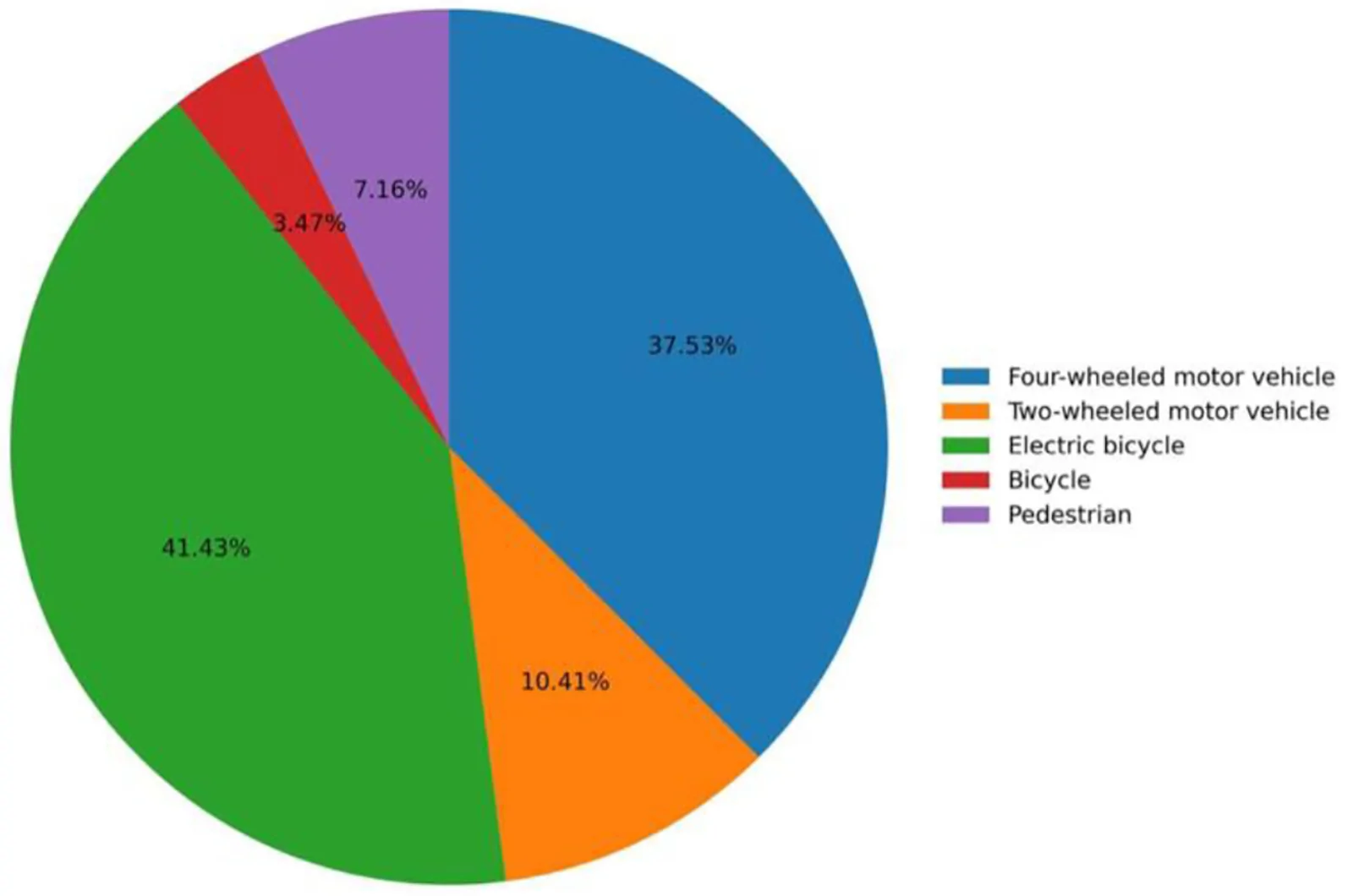
Distribution of traffic accident categories in TSCI. TSCI = traumatic spinal cord injury.

Among the 200 patients with NTSCI, neoplasm was the leading cause (33.00%, 66/200), followed by demyelinating disease (20.00%, 40/200), infectious disease (17.00%, 34/200), vascular disease (15.50%, 31/200), and degenerative disease (14.50%, 29/200; Table 2).

### 3.4. Neurological severity and injury level

Neurological profiles differed substantially between groups. In TSCI, incomplete tetraplegia was the most frequent phenotype (58.38%, 662/1134), followed by complete tetraplegia (26.90%, 305/1134), incomplete paraplegia (7.50%, 85/1134), and complete paraplegia (7.23%, 82/1134). In NTSCI, incomplete paraplegia predominated (59.00%, 118/200), followed by incomplete tetraplegia (35.50%, 71/200), complete paraplegia (4.50%, 9/200), and complete tetraplegia (1.00%, 2/200; Table 2). AIS grade distributions differed between TSCI and NTSCI (*P* < .001): AIS A accounted for 34.13% (387/1134) of TSCI but only 5.50% (11/200) of NTSCI (Table 1 and Table S2, Supplemental Digital Content 2).

Regarding injury level, cervical cord injury was the predominant category in TSCI (84.04%, 953/1134), whereas thoracic cord injury (28.00%, 56/200), cervical cord injury (25.00%, 50/200), and lumbar cord injury (24.50%, 49/200) were more evenly represented in NTSCI. Multilevel cervicothoracic and thoracolumbar injuries accounted for 11.50% (23/200) and 11.00% (22/200) of NTSCI, respectively. Injury-level distributions differed between groups (*P* < .001; Table 1 and Fig. 2).

**Figure 2.**
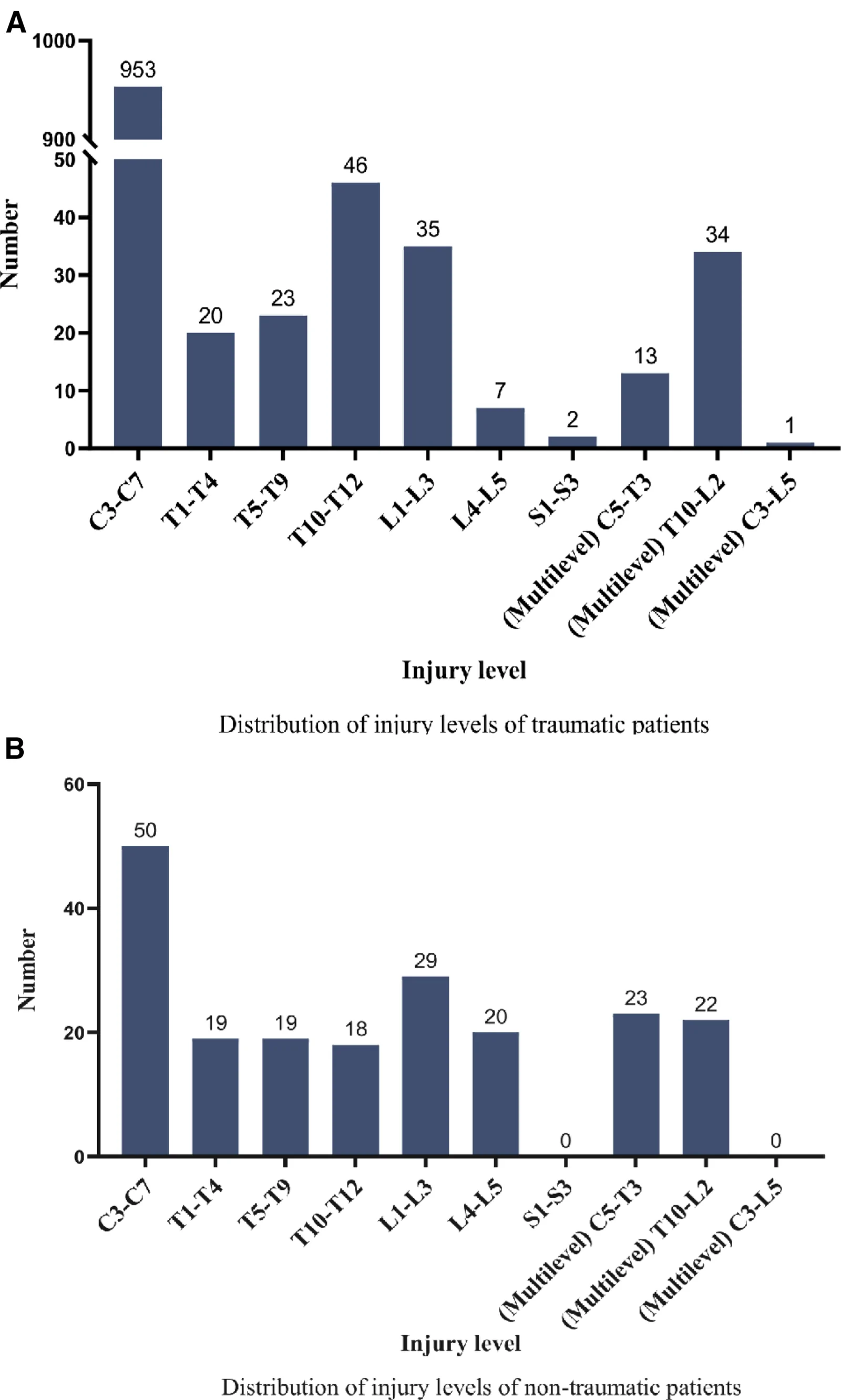
Distribution of injury levels in (A) TSCI and (B) NTSCI. NTSCI = nontraumatic spinal cord injury, TSCI = traumatic spinal cord injury.

### 3.5. Treatments and LOS

Overall, 84.26% (1124/1334) of patients underwent surgery. Surgical treatment was more frequent in TSCI than NTSCI (88.98%, 1009/1134 vs 57.50%, 115/200; *P* < .001). Hyperbaric oxygen therapy was also more frequent in TSCI (21.34% vs 13.50%; *P* = .011), whereas medication use was more frequent in NTSCI (21.00% vs 13.49%; *P* = .006). Rehabilitation therapy, traditional therapy, and assistive device use did not show statistically significant between-group differences (Table 3). LOS was markedly right-skewed. Median LOS was 12 days (IQR = 8–21) for TSCI and 46 days (IQR = 33.75–61) for NTSCI (*P* < .001). For complete injuries (AIS A), median LOS was 18 days (IQR = 11–28) in TSCI and 74 days (IQR = 66.5–89.5) in NTSCI (*P* < .001); for incomplete injuries (AIS B–D), corresponding values were 10 days (IQR = 7–18) and 44 days (IQR = 33–58), respectively (*P* < .001). All verified LOS values were retained in the analysis.

**Table 3:** Treatment patterns and length of stay among patients with SCI.

Variable	TSCI (n = 1134)	NTSCI (n = 200)	Overall (n = 1334)	*P* value
Surgery				<.001
Yes	1009 (88.98%)	115 (57.50%)	1124 (84.26%)	
No	125 (11.02%)	85 (42.50%)	210 (15.74%)	
Rehabilitation therapy				.114
Yes	746 (65.78%)	143 (71.50%)	889 (66.64%)	
No	388 (34.22%)	57 (28.50%)	445 (33.36%)	
Traditional therapy				.883
Yes	664 (58.55%)	116 (58.00%)	780 (58.47%)	
No	470 (41.45%)	84 (42.00%)	554 (41.53%)	
Hyperbaric oxygen therapy				.011
Yes	242 (21.34%)	27 (13.50%)	269 (20.16%)	
No	892 (78.66%)	173 (86.50%)	1065 (79.84%)	
Assistive devices				.096
Yes	445 (39.24%)	91 (45.50%)	536 (40.18%)	
No	689 (60.76%)	109 (54.50%)	798 (59.82%)	
Medication				.006
Yes	153 (13.49%)	42 (21.00%)	195 (14.62%)	
No	981 (86.51%)	158 (79.00%)	1139 (85.38%)	
Length of stay, d, median (IQR)				
Complete injury (AIS A)	18 (11–28)	74 (66.5–89.5)	18 (11–31)	<.001
Incomplete injury (AIS B–D)	10 (7–18)	44 (33–58)	13 (8–28.25)	<.001
Overall	12 (8–21)	46 (33.75–61)	15 (9–29)	<.001

Treatment variables are not mutually exclusive. Treatment *P* values were calculated using Pearson chi-square tests.

LOS is reported as median (IQR) because of marked right-skewness. LOS comparisons used two-sided Mann–Whitney *U* tests.

Complete injury = AIS A; incomplete injury = AIS B to D. No LOS values were missing.

AIS = American Spinal Injury Association Impairment Scale, IQR = interquartile range, LOS = length of stay, NTSCI = nontraumatic spinal cord injury, SCI = spinal cord injury, TSCI = traumatic spinal cord injury.

### 3.6. In-hospital complications

A primary in-hospital complication was recorded in 1048 of 1334 patients (78.56%): 888/1134 (78.31%) with TSCI and 160/200 (80.00%) with NTSCI (*P* = .591). The overall distribution of the 6 primary complication categories plus no recorded primary complication differed between groups (*P* = .040). Urinary tract infection was more frequent in TSCI than NTSCI (24.78% vs 16.00%, *P* = .007), whereas pulmonary infection was more frequent in NTSCI (18.00% vs 12.26%, *P* = .027). Hyponatremia, pressure ulcers, deep vein thrombosis, and other primary complications did not show statistically significant between-group differences (Table 4).

**Table 4:** Primary in-hospital complications among patients with SCI.

Primary in-hospital complication	TSCI (n = 1134)	NTSCI (n = 200)	Overall (n = 1334)	*P* value
Any recorded primary complication	888 (78.31%)	160 (80.00%)	1048 (78.56%)	.591
Urinary tract infection	281 (24.78%)	32 (16.00%)	313 (23.46%)	.007
Pulmonary infection	139 (12.26%)	36 (18.00%)	175 (13.12%)	.027
Hyponatremia	128 (11.29%)	27 (13.50%)	155 (11.62%)	.368
Pressure ulcers	165 (14.55%)	37 (18.50%)	202 (15.14%)	.151
Deep vein thrombosis	82 (7.23%)	15 (7.50%)	97 (7.27%)	.893
Other complications	93 (8.20%)	13 (6.50%)	106 (7.95%)	.412
No recorded primary complication	246 (21.69%)	40 (20.00%)	286 (21.44%)	.591

One primary in-hospital complication was recorded per patient; complication categories were mutually exclusive. The 6 specific categories sum to the number of patients with any recorded primary complication (n = 1048).

Individual-row *P* values compare TSCI and NTSCI using Pearson chi-square tests. The overall distribution of the 6 primary complication categories plus no recorded primary complication differed between groups (*P* = .040).

NTSCI = nontraumatic spinal cord injury, SCI = spinal cord injury, TSCI = traumatic spinal cord injury.

## 4. Discussion

Using 10 years of inpatient data from 2 tertiary centers in Jinan, this study directly compared the hospital-based profiles of TSCI and NTSCI. TSCI constituted the majority of admissions, was more strongly male-predominant, and was characterized primarily by falls and traffic-related injuries. NTSCI accounted for a smaller proportion of admissions but showed a heterogeneous etiologic spectrum and substantially longer hospitalization. Neurological severity, injury-level distribution, treatment patterns, and the distribution of primary in-hospital complication categories also differed between groups. Because these comparisons were unadjusted, they should be interpreted as differences in observed case mix rather than as independent effects of traumatic versus nontraumatic etiology.

The older age structure of the TSCI cohort and the prominent contribution of low falls are consistent with reports from aging societies and other settings in which low-energy falls contribute substantially to TSCI,^[9,12]^ as well as with Chinese hospital-based cohorts reporting a high proportion of fall-related injuries.^[10,13]^ Traffic-related injuries nevertheless remained common, and electric bicycles represented the largest traffic-related category in the present cohort. These findings support targeted prevention priorities within the local setting, particularly fall-risk reduction among older adults and safer road behaviors for electric-bicycle and motor-vehicle users. Community- and family-level education and modifiable fall-risk interventions may be relevant components of such strategies.^[14–16]^ However, because this study did not include a population denominator or exposure data, the observed hospital case mix should not be interpreted as a population incidence estimate or as a direct measure of regional disease burden.

Neurological profiles also differed between groups. Cervical cord involvement predominated in TSCI, consistent with other Chinese series,^[10,13]^ whereas NTSCI showed a more dispersed injury-level distribution. Incomplete injury predominated in NTSCI, while AIS A was substantially more frequent in TSCI. These observations are descriptive and may reflect differences in injury mechanism, etiology, referral patterns, and hospital admission pathways. Although prehospital care, emergency triage, and timing of surgery are clinically important in acute SCI,^[17,18]^ these factors were not measured in the present study; therefore, the comparatively high proportion of AIS A in TSCI should be viewed as a finding that warrants further investigation rather than evidence of deficiencies in any specific component of care.

NTSCI patients had a markedly longer unadjusted LOS than TSCI patients. This pattern may be related to differences in etiology, neurological severity, treatment requirements, referral pathways, or other baseline characteristics rather than to nontraumatic etiology itself. Similarly, although the overall distribution of primary in-hospital complication categories differed between groups, the proportion of patients with any recorded primary complication was similar. Urinary tract infection was more frequent in TSCI, whereas pulmonary infection was more frequent in NTSCI; pressure ulcers, hyponatremia, deep vein thrombosis, and other primary complications did not differ significantly. These findings may help identify practical areas for inpatient surveillance and supportive care, including bladder management, respiratory complication prevention, skin care, and early rehabilitation engagement.^[19]^

Strengths of this study include a decade-long dual-center inpatient dataset, direct comparison of TSCI and NTSCI within the same regional tertiary-care setting, and assessment across multiple clinical dimensions. Several limitations should be acknowledged. First, because this was a hospital-based study from 2 tertiary referral centers, the sample is susceptible to referral and selection bias and cannot be used to estimate population-level incidence. Second, the retrospective design may be vulnerable to information and classification bias. Third, the TSCI and NTSCI groups differed markedly in age distribution, neurological severity, etiology, treatment, and injury level, and no multivariable adjustment was performed; consequently, observed LOS and complication differences cannot be attributed independently to traumatic versus nontraumatic etiology. Fourth, the database captured only 1 primary in-hospital complication per patient, so concurrent or secondary complications were not systematically represented. Fifth, long-term follow-up data were unavailable, precluding assessment of long-term functional outcomes. Finally, the NTSCI sample was relatively small, limiting precision for some stratified descriptions. Multicenter prospective registries with longitudinal follow-up are needed to more accurately characterize regional case mix and evaluate prevention and care-pathway strategies.^[20,21]^

## 5. Conclusion

Within 2 tertiary hospitals in Jinan, TSCI and NTSCI showed different demographic, etiologic, neurological, treatment, hospitalization, and primary complication patterns. TSCI was characterized by prominent fall- and traffic-related injury mechanisms, including a substantial contribution from electric bicycle-related events, whereas NTSCI showed heterogeneous etiologies and substantially longer hospitalization. These findings may support targeted prevention priorities and differentiated hospital care pathways. Because the analyses were hospital-based and unadjusted, the observed patterns should not be interpreted as population-level incidence estimates or independent causal effects. Future multicenter prospective registries with longitudinal follow-up are warranted.

## Author contributions

**Formal analysis:** Min Zhao, Hezi Chang.

**Investigation:** Xiaolin Chen.

**Methodology:** Yingrun Fu.

**Resources:** Xiaolin Chen.

**Software:** Ruoxian Song.

**Supervision:** Ruoxian Song.

**Writing – original draft:** Linyang Song, Hezi Chang, Yingrun Fu.

**Writing – review & editing:** Linyang Song, Kun Wang, Hezi Chang, Yingrun Fu.




