# Supplementary material for: Current epidemiological profile and characteristics of traumatic and nontraumatic spinal cord injury in Jinan, China: A two-center retrospective observational study
Source: Medicine (Baltimore). 2026 Nov 6;105(45):e51397. doi: 10.1097/MD.0000000000051397 (PMC13747360; doi:10.1097/MD.0000000000051397)
Supplement: Supplementary file 1 [file medi-105-e51397-s001.docx]

**Supplementary Table S1. Traumatic etiology according to age group**

| **Age, years** | **Traffic accidents** | **Low fall** | **High fall** | **Machinery-related injury** | **Sport** | **Falling objects** | **Total** |
| --- | --- | --- | --- | --- | --- | --- | --- |
| 0-15 | 8 (22.86%) | 5 (14.29%) | 11 (31.43%) | 2 (5.71%) | 8 (22.86%) | 1 (2.86%) | 35 (100.00%) |
| 16-30 | 22 (40.74%) | 6 (11.11%) | 17 (31.48%) | 2 (3.70%) | 4 (7.41%) | 3 (5.56%) | 54 (100.00%) |
| 31-45 | 37 (35.58%) | 16 (15.38%) | 34 (32.69%) | 1 (0.96%) | 6 (5.77%) | 10 (9.62%) | 104 (100.00%) |
| 46-60 | 96 (42.86%) | 41 (18.30%) | 51 (22.77%) | 7 (3.12%) | 10 (4.46%) | 19 (8.48%) | 224 (100.00%) |
| 61-75 | 161 (42.71%) | 108 (28.65%) | 64 (16.98%) | 9 (2.39%) | 18 (4.77%) | 17 (4.51%) | 377 (100.00%) |
| ≥76 | 137 (40.29%) | 117 (34.41%) | 52 (15.29%) | 5 (1.47%) | 21 (6.18%) | 8 (2.35%) | 340 (100.00%) |
| Overall | 461 (40.65%) | 293 (25.84%) | 229 (20.19%) | 26 (2.29%) | 67 (5.91%) | 58 (5.11%) | 1134 (100.00%) |

Percentages are calculated within each age group; the Overall row uses TSCI n=1,134 as the denominator.
