# Supplementary material for: Current epidemiological profile and characteristics of traumatic and nontraumatic spinal cord injury in Jinan, China: A two-center retrospective observational study
Source: Medicine (Baltimore). 2026 Nov 6;105(45):e51397. doi: 10.1097/MD.0000000000051397 (PMC13747360; doi:10.1097/MD.0000000000051397)
Supplement: Supplementary file 2 [file medi-105-e51397-s002.docx]

**Supplementary Table S2. AIS grade according to non-traumatic etiology**

| **AIS grade** | **Demyelinating disease** | **Neoplasm** | **Degenerative disease** | **Vascular disease** | **Infectious disease** | **Overall** |
| --- | --- | --- | --- | --- | --- | --- |
| A | 1 (2.50%) | 2 (3.03%) | 2 (6.90%) | 1 (3.23%) | 5 (14.71%) | 11 (5.50%) |
| B | 7 (17.50%) | 13 (19.70%) | 10 (34.48%) | 7 (22.58%) | 9 (26.47%) | 46 (23.00%) |
| C | 17 (42.50%) | 24 (36.36%) | 10 (34.48%) | 11 (35.48%) | 11 (32.35%) | 73 (36.50%) |
| D | 15 (37.50%) | 27 (40.91%) | 7 (24.14%) | 12 (38.71%) | 9 (26.47%) | 70 (35.00%) |

Percentages are calculated within each NTSCI etiology; the Overall column uses NTSCI n=200 as the denominator. AIS, American Spinal Injury Association Impairment Scale.
